# Supplementary material for: Are carbon emissions trading and green financial instruments synergistic? -Comprehensive quantitative research based on content analysis
Source: PLoS One. 2024 Mar 7;19(3):e0298601. doi: 10.1371/journal.pone.0298601 (PMC10919601; doi:10.1371/journal.pone.0298601)
Supplement: S1 Data — (ZIP) [file pone.0298601.s001.zip › Data/地级市Z_F工作报告.zip!/╔╜╬≈/╠1⁄2╘¡╩╨/└┤╫╘╣1⁄2╓┌║┼í╛┬φ┐╦╩2╛▌═°í┐_2018.docx]

各位代表：

现在，我代表市人民政府，向大会报告工作，请予审议，并请政协委员和列席人员提出意见。

一、2017年工作回顾

过去一年，全市上下以习近平新时代中国特色社会主义思想为指导，认真贯彻落实党的十九大精神和习近平总书记视察山西重要讲话精神，牢固树立新发展理念，坚持向改革开放要动力，向创新驱动要活力，向转型升级要竞争力，奋力实现“两个走在前列”，较好完成全年经济社会发展主要目标任务。

——经济运行持续稳定增长。2017年完成地区生产总值3382.18亿元，增长7.5%；规模以上工业增加值631.23亿元，增长9.0%；固定资产投资964.86亿元，增长6.8%；一般公共预算收入311.85亿元，增长10.3 %；社会消费品零售总额1767.82亿元，增长6.1%；外贸进出口总额915.25亿元，增长4.1%；城镇常住居民人均可支配收入31469元，增长6.2%；农村常住居民人均可支配收入15595元，增长6.9%。

——供给侧结构性改革取得初步成果。着力发展实体经济，加快工业强市步伐。江铃重汽、比亚迪新能源汽车、太钢碳纤维二期、阳煤太化新材料、太重地铁盾构机制造等相继投产，富士康手机智能制造和全球维修持续发力，产值突破700亿元。加快发展现代都市农业，调整种植结构，优化养殖布局，培育九牛、宝迪等12家农业产业化龙头企业。现代服务业发展势头良好。阳光城洲际、富力铂尔曼等五星级酒店、传化智能物流、华宇百花谷综合体等相继运营，华润万象城、新城吾悦商业、中海、信达超高层商务楼宇建设进展顺利。科技创新能力不断提高。新兴产业和现代服务业对经济增长的贡献进一步增强。

——城市基础设施建设和管理不断提升。东中环北延、新店街、北沙河、玉门河等八河道路快速化改造，迎泽大街下穿火车站、双塔南路、东峰路北延等110余条、200余公里城市道路改造建设全面推进。地铁2号线完成10个车站主体结构，1号、3号线前期工作顺利。高铁南站、太原站东广场改造启动。完成26个城中村50多个棚户区总计1000余万平方米拆迁，滨河东西路、龙城大街、阳兴大道等综合整治、绿化提升成效明显。晋源东区地下综合管廊、餐厨垃圾处理、晋阳污水处理投入运行。交通秩序整治取得初步成效。

——环境质量改善迈出新步伐。持续推进集中供热全覆盖，新增供热面积3300万平方米。“煤改电”“煤改气”完成11.6万户，市区严格禁煤。关停一电厂，取缔整治散乱污企业。淘汰老旧机动车、黄标车5000辆。实行领导包联责任制，“秋冬防大气污染综合治理”攻坚行动成效明显，空气质量指数同比下降28.7%，PM2.5浓度均值同比下降33.1%，重污染天数同比下降75%。全面推行河长制，实施八河治理，雨污分流、河道复清、两岸增绿、城市整治协同推进。汾河三期水利主体工程完成。南寒体育公园、昌宁公园等建成开放，晋阳湖景区、太原植物园建设和迎泽公园改造进展顺利。全市共实施绿化面积580.15万平方米，完成营造林42.6万亩，建成区绿化覆盖率、绿地率、人均公园绿地面积分别达42.19%、37.18%和12.18平方米。

——民生保障建设持续推进。太原五中、成成中学、一外、二外和中心医院、人民医院、妇幼保健院、儿童福利院、康宁医院等总投资超百亿元的民生工程完成主体。棚户区改造新开工3.25万套、基本建成3.36万套。医药卫生体制改革成效明显。城乡居民养老、医疗、失业等五项保险全覆盖。新建社区养老服务中心64个，老年日间照料中心87个。城镇登记失业率3.41%。完成73个贫困村22069人脱贫任务，阳曲县脱贫摘帽进入公示验收阶段。“双拥”工作不断推进，文明城市创建迈出新步伐。积极化解信访疑难问题，安全生产形势总体平稳。

——文化事业和文化产业加快发展。持续推进晋祠景区提升完善，青龙古镇、太原古县城、太山龙泉寺、天龙山大景区、双塔景区保护性改造提升，华夏文明主题公园建设。府城普光寺、文殊寺、五一路历史片区等保护性修复基本完成。市图书馆改扩建竣工开放。晋剧《关公》入选2017年度国家艺术基金资助项目，电视剧《于成龙》荣获中宣部“五个一工程”奖。体育事业不断发展，我市运动员在第十三届全国运动会上取得优异成绩。“担复兴大任，做时代新人”大型讲述活动广获好评。

——政府自身建设和民主法治建设全面加强。自觉接受人大依法监督、政协民主监督，认真听取各民主党派、工商联、无党派人士建言献策，办理人大代表建议385件、政协提案532件，办复率100%。“放管服效”改革扎实推进，承接国务院、省政府取消下放行政职权52项，下放31项。“五规合一”信息平台上线运行。新政务服务中心全面启用，行政审批事项全部入驻办事大厅。深入开展“两学一做”学习教育，持之以恒纠正“四风”。坚决查处和纠正违法违规行为，堵塞工程招投标、土地出让等重要环节的漏洞，弘扬忠诚、干净、担当的政风。

各位代表，过去一年所取得的成绩，是高举习近平新时代中国特色社会主义思想伟大旗帜，在省委、省政府和市委的坚强领导下，在市人大、市政协和社会各界有效监督、大力支持下，全市广大干部群众上下同心、团结奋斗的结果。在此，我代表市人民政府，向全市人民，向各位人大代表和政协委员，向驻并部队和公安民警，向所有关心支持太原改革发展的各界朋友，表示崇高的敬意和衷心的感谢！

在肯定成绩的同时，我们也清醒认识到，我市发展仍面临不少困难和挑战，政府工作还有不少问题和不足。集中表现为：经济总量不大和质量不高并存，发展不平衡不充分的问题还较为突出；战略性新兴产业发展不足，大项目、好项目、新项目不多，传统产业改造提升不快，创新驱动、转型升级面临严峻挑战；改革力度不大，办事效率不高，服务质量不优，营商环境不良的问题依然存在；基础设施欠账较多，管理水平落后，城市环境质量改善形势不容乐观；住房、教育、医疗等民生事业发展不均衡、质量不高问题仍然突出；一些领域不正之风和腐败问题仍有发生。对此，我们要本着对人民高度负责的精神，坚持问题导向，强化使命担当，采取有力举措，切实加以解决。

二、2018年工作安排

今年是贯彻党的十九大精神的开局之年，是改革开放40周年，是决胜全面建成小康社会、实施“十三五”规划承上启下的关键一年。今年政府工作的总体要求是：以习近平新时代中国特色社会主义思想为指导，全面贯彻党的十九大和十九届二中、三中全会精神，按照“两个走在前列”的目标要求和“五个扎实”的工作要求，认真落实市委十一届三次、四次全会精神，统筹推进“五位一体”总体布局，协调推进“四个全面”战略布局，坚持稳中求进工作总基调，坚持把深化供给侧结构性改革与深化转型综改试验区建设有机结合起来，充分发挥转型综改示范区的引领带动作用，全面推进稳增长、促改革、调结构、惠民生、防风险各项工作，推动质量变革、效率变革、动力变革，在打好防范化解重大风险、精准脱贫、污染防治攻坚战方面取得扎实进展，促进经济社会持续健康发展，进一步提升在全省的首位度和在全国省会城市的综合排名，奋力谱写文明开放富裕美丽太原新篇章。

主要预期指标是：地区生产总值增长8%左右，规模以上工业增加值增长10%，固定资产投资增长10%，社会消费品零售总额增长7.5%，一般公共预算收入增长7%，城乡常住居民人均可支配收入分别增长6.5%和6.5%以上，居民消费价格涨幅控制在3%左右，城镇登记失业率控制在4%以内，约束性指标不折不扣完成省下达任务。

（一）以转型项目建设年为重要契机，深入推进供给侧结构性改革

进入新时代，我国社会主要矛盾发生了关系全局的历史性变化，高质量发展是必须跨越的关口。新时代带来新机遇，新要求提出新挑战。必须抓住难得的历史机遇，以转型项目建设年为抓手，加快转变发展方式，优化产业结构，转换增长动能，把实体经济做实做强做优。

强力推进转型升级，发展壮大新动能。充分发挥综改示范区新引擎的核心功能，打好转型综改攻坚战。综改示范区肩负着“示范区”“排头兵”和“新高地”的重大使命。要抓住重点，突出特色，明确主攻方向，集中优势资源，构建布局结构优、规模体量大、延伸配套好、支撑带动强的现代产业集群。要打造信息技术产业集群，完善富士康苹果手机整机制造和全球维修中心，迈上产值800亿元新台阶。加快中科院超算中心、山西移动云计算数据中心、山西浪潮大数据产业园等项目建设。要打造先进装备制造产业集群，倾力支持江铃集团做强重卡、开发中卡，引进天津宇傲、江苏长凯、苏州万隆等关键零部件配套企业，形成产业集群。推进磁浮轨道交通、晋西轨道交通等项目建设。要打造新材料产业集群，加快中电科碳化硅、银邦金属材料等重点项目建设。要打造新能源产业集群，推进比亚迪新能源客车、动力电池组装、专用重卡制造。加快西安中科镁基电池、山西移动能源产业等项目建设。

发挥老工业基地优势，推进传统产业提质增效。把资源存量与先进技术突破有机结合起来，发挥好产业转型的战略优势。推进太钢冷轧取向硅钢、T800碳纤维三期、不锈钢深加工产业集群、铝镁合金基地、中晋交能大数据等转型项目，阳煤太化新材料延伸产品尼龙6、尼龙66，己内酰胺10万吨技改扩产、太重地铁装备制造等新项目建设。加快推进清徐煤焦化工高端化、智能化、集群化、绿色化循环园区建设，紧跟世界能源技术革命新趋势，推进煤炭清洁高效利用，打造能源革命新样板。大力发展工业互联网，深入实施“中国制造2025”，创造互联网、大数据、人工智能和实体经济深度融合的新载体、新模式、新业态。发挥军工产业集聚优势，推进电子信息科技、电子智能制造等20个项目落地建设，加快形成全要素、多领域、高效益的军民融合深度发展格局，争创国家级军民融合创新示范区。

加快发展现代服务业，培育新的经济增长点。大力发展现代物流、会展经济、咨询服务、科技服务、金融服务等生产性服务业。推进太原国际会展中心、苏宁广场、红星美凯龙爱琴海、远大购物、银泰中心、丽思卡尔顿六星级酒店、悦榕庄五星级酒店等项目开工建设。基本完成占地2000亩动物园升级扩容改造，集动物观赏、儿童游乐、智慧教育于一体，力争跻身全国一流行列。华润万象城、阳光皇冠假日8月初实现对外运营。发展以“互联网+服务”为代表的新型服务业，带动服务业整体升级。

用好改革开放“关键一招”，构建推动经济高质量发展体制机制

改革开放是发展进步的活力之源，是赶上时代前进步伐的重要法宝。要以更宽广的视野、更高的目标要求、更有力的举措，突出重点领域和关键环节，坚持“改革不停顿、开放不止步”，将深化改革扩大开放进行到底。

深化“放管服效”改革，全力营造“六最”营商环境。全面实施市场准入负面清单制度，深化“证照分离”改革，重点是照后减证，能减则减，能合则合，大幅压缩企业开办时间。全面推行企业投资项目承诺制，落实统一清单告知，统一平台办理，统一流程再造，统一多图联审，统一收费管理，变先批后建为先建后验，变事前审批为事中事后监管服务，为市场主体添活力，进一步解放和发展生产力。深入推进“互联网+政务服务”，推进民生服务事项网上办理，破繁苛之弊，兴便民之利，让人民群众共享大数据服务的便捷。深化综合执法改革，根治多头多层重复执法问题。推进简政放权，把该放的权力放下去，把该管的事情管好。

全面推进体制机制创新，提高资源配置效率效能。深化国资国企改革，制定出资人监管权责清单，完善有效制衡的法人治理结构，完成“三供一业”分离，持续瘦身健体，提升主业核心竞争力。建立多主体供应、多渠道保障、租购并举的住房制度。推进农村集体产权制度改革。深化财税体制改革，提高财力使用效率。完善产权制度和要素市场化配置机制，推动创新要素自由流动和聚集，资源向优质企业和产品集中，以优质的制度供给、服务供给和要素供给，增强企业的核心竞争力，打造高质量发展的强大动能。

推进民营经济改革发展，营造公平竞争的市场环境。产权激励是最大激励，是民营企业的恒心所在。要坚持“两个毫不动摇”，落实好中央和省、市一系列支持非公经济发展的政策举措，在透明的法治环境下保护好产权，破除信贷、上市、创新、招投标、人才等方面的歧视性政策限制和各种隐性障碍，多渠道破除融资难、融资贵问题。加强对中小企业创新支持，培育更多具有自主知识产权和核心竞争力的创新型企业。促进大众创业、万众创新上水平，注重发现和培育有发展前景的“双创”企业，拓展市场化专业化众创空间，高质量建设“双创”基地城市示范。构建“亲”“清”新型政商关系，营造尊重、激励和保护企业家干事创业的社会氛围。

推动形成全面开放新格局，以开放促改革、促发展。要站在新的历史起点上，推动思想再解放，改革再深化，开放再扩大。开放需要海纳百川的胸襟，更需要招凤引凰的魅力。要认真落实市委制定的人才激励政策，吸引国际国内人才流、资金流、信息流汇聚，为创新驱动提供人才智力支撑。继续加大招商引资力度，着眼招大引强，紧盯名企名品名家，聚焦优秀企业，支撑有效投资、转型跨越。用好用足国务院批准太原建设国家可持续发展议程创新示范区的政策支持，强化科技创新、转型升级、绿色发展，创造资源型城市转型发展可复制、可推广的现实样板。加强同中科院、同济、清华、交大等科研院所和高校的合作，支持富士康并州研发中心，启动一批科技创新重大项目，推进智能制造研发，加快创新成果转化应用，不断增强经济创新力和竞争力。

塑造文化产业精品，发展文化旅游支柱产业

文化是凝魂聚气、强基固本的基础，是一个城市创造力、影响力和竞争力的重要源泉。要弘扬中华优秀传统文化，发展社会主义先进文化，为全市经济社会发展提供坚强的思想保证、强大的精神力量、丰润的道德滋养。

历史是文化的载体，文化是历史的血脉。太原拥有2500多年的建城文明史，丰厚的历史土壤，留下了宝贵的物质文化遗产和精神文化遗产。要本着对历史、对人民、对未来负责的精神，努力实现传统文化的创造性转化、创新性发展。要聚焦发力，全面完成太山龙泉寺、天龙山石窟、太原明代古县城、双塔景区、青龙古镇和千年府衙等六大历史文化遗产保护性修复，完善晋祠景区提升改造，初步完成太化工业文明遗址保护利用，配套道路建设、环境综合治理、优质服务供给，积极创建5A级景区。让陈列在大地上不可再生的文化遗产、收藏在博物馆里的文物都活起来，再现昔日辉煌，擦亮城市名片，坚定朝着建设文化旅游目的地的战略目标迈进。

着力培育文化市场主体，打造现代文化旅游精品景区。华强华夏文明主题公园年内竣工开业，将成为我市文化旅游业一张靓丽名片。远洋太古里植入双塔景区，古今文化经典相融，打造城市文化广场和城市客厅。建设国际一流、富有特色的自行车赛道，集体育竞技、文化旅游、国际交流、产业创新于一体，形成具有世界影响力的城市品牌。晋阳湖公园引进优秀团队，创作高科技唯美水秀和国际演艺，塑造影响深远的文化精神标识，提升太原文化的吸引力和竞争力。

大力发展文化事业，繁荣文艺创作，提高文化软实力。要坚持把中国精神作为社会主义文化的灵魂，创作更多反映时代呼声、展现人民奋斗、陶冶高尚情操的优秀作品，讲好太原故事。要把晋剧作为“国粹”精心呵护、倾力支持，加强艺术传承、人才培养、精品创作。加快建设晋剧院、话剧院，让艺术进入高雅的殿堂。支持太原莲花落、杂技风火流星等非物质文化遗产薪火相传，不断焕发新的生机和风采。培育新型文化业态，加强对外文化交流。推进传统媒体与新兴媒体深度融合。加强国防教育，强化全民安不忘危、和不忘战的国防意识。继续实施“送戏下乡”、公益电影下乡等文化惠民工程，提升农村公共文化服务水平。

加强文物保护和文创产品合作研发，推进互联网与文物宣传创新深度融合。加强晋阳古城遗址考古研究，科学展示遗址成果。加强与故宫博物院文物展示、文创产品、文物保护等方面深度合作，推动文博事业进入全国一流水平。做好府城历史片区保护，加快皇庙修复、文庙周边环境整治提升。

（四）以承办全国二青会为动力，全面提升省会城市整体形象和文明水平

城市化是现代化的必由之路。建设好省会城市要认识、尊重、顺应城市发展规律，坚持创新、协调、绿色、开放、共享的发展理念，让人民群众在城市生活得更方便、更舒心、更幸福、更美好，提高太原都市区国际化、现代化、智能化水平。

以更加富有前瞻性的规划引领城市发展。规划是城市不败的基础，城市以规划论输赢。坚持不断学习、借鉴、完善，用最先进的发展理念和国际一流水准规划设计建设，下功夫做好单体建筑设计，打造城市标杆精品和典范，经得起历史检验。要着眼精彩、非凡、超越，规划承办好二青会，努力开创太原发展更加美好的明天。

持续推进城市基础设施建设。高标准完成西中环南延、东中环北延、南中环东延、龙城大街东延、滨河东路南延及3座汾河大桥、滨河西路北延、大运路等快速路建设，全面完成八河快速路建设，进一步完善提升城市快速路网。推进迎泽大街东延、学府街东延、马练营路北延、龙城南北街、军民路等城市主次干道和小街小巷改造建设。加快地铁2号线建设，推进1号、3号线建设。完成高铁南站、太原站东广场建设，建立高进低出快速交通综合服务体系。开工建设东南客运站、西北二环高速路及娄烦高速连接线。推进海绵城市建设，加快晋源东区、综改示范区综合管廊建设。完成南坪头、马庄、五六号缓洪池等上游防洪调蓄工程。推进500千伏太原北等重大电网工程。

全面提升城市管理水平。以城市管理现代化为指向，加快智慧城管、智慧交通建设，充分运用互联网大数据，科学、精准、高效调度管理。深化城市管理体制改革，坚持以人为本、源头治理、权责一致、协调创新的原则，推进城市综合执法，建立权责明晰、服务为先、管理优化、执法规范、安全有序的城市管理体制。要针对交通节点堵塞及各种乱象，精准施策，从严管理，综合整治，一件一件抓到底，抓出新成效，治出新水平。

全力做好二青会各项筹备工作。二青会是我省第一次主办的全国大型综合性运动会。要加快青运村和山西国际体育交流中心、水上运动中心、沙滩排球场、排球馆、足球场等场馆建设，推进滨河体育中心、网球中心等场馆改造，确保所有项目年底竣工。要高质量、高水平承办好全省第十五届运动会，作为迎接二青会的“带妆彩排”、预备检阅。要全面提高服务业水平，全面整治美化城市立面，亮化城市，增绿补绿，让城市更靓丽，让人民更幸福。

（五）推进生态文明建设，打赢蓝天保卫战

生态环境是最普惠的民生福祉。坚持生态优先、绿色发展，是发展观的一场深刻革命。要以提高生态环境质量为目标，综合系统治理环境质量突出问题，努力建设蓝绿交织、清新明亮、山水相融的美丽太原。

严控大气污染。推进清洁供热全覆盖，新增集中供热2000万平方米。城南、城西两个燃煤热源厂改造为燃气供热。城区燃煤锅炉清零。三县一市全部淘汰20吨及以下燃煤锅炉，完成10万户农村清洁供暖改造。深度治理工业企业污染，钢铁、化工、水泥等要达到特别排放限值要求。持续开展“散乱污”企业整治和挥发性有机物治理。严格落实建筑工地扬尘治理“六个百分之百”要求。加大面源污染防治监管，严禁秸秆焚烧和露天烧烤，综合整治渣土消纳场所。推进货车运营清洁化。强化网格监管、靶向攻坚，精准施策、科学治霾。

加大水污染防治。实施水源保护工程，保障汾河水库水质稳定达标。全面落实河长制，加大汾河水源地保护，完成汾河三期治理工程，建设人工湿地。加快汾东和城南35万吨污水处理厂建设，水西关、康乐街和60余条小街小巷雨污分流工程，从源头上消除黑臭水体，让母亲河“水量丰起来、水质好起来、风光美起来”。

加强土壤污染治理。建立污染土地动态清单和联动监管机制，着力解决影响农产品安全和人居环境健康两大突出问题。做好生活垃圾分类试点工作，加快循环经济环卫产业示范基地建设，生活垃圾焚烧电厂、市政污泥与污水处置项目、餐厨垃圾处置二期项目建成投运，推进垃圾减量化、无害化、资源化。

持续推进生态建设。加快东西北山造林绿化提档升级和市区园林绿化，完成营造林30万亩。初步完成11.7平方公里晋阳湖大生态体系建设，构建“三面环山、一水中分、九河环绕、一湖点睛”水韵龙城格局。完成迎泽公园、南寨公园、双塔公园、牛驼寨公园扩容提质改造，基本完成植物园建设，新改扩建天龙山国家森林公园、环城森林公园和一批街头游园。加大道路两侧、南站沿线、八河沿边、城市裸露面绿化力度，加快山体破坏面生态修复，大幅扩大绿色生态空间，让绿色成为太原的底色。建成区绿化覆盖率、绿地率均增加0.5个百分点。

（六）树立以人民为中心的发展思想，推进各项社会事业发展

民生连着民心，民心是最大的政治。要以民评民说为标准、民心民力为依靠、民意民声为动力、民思民盼为方向、民惠民富为目标，用心用情用力做好保障改善民生各项工作。

打好精准脱贫攻坚战。阳曲县作为省定贫困县，实现脱贫攻坚首战告捷。要继续提高质量、巩固成果、夯实基础。娄烦县是我市唯一国定贫困县，今年要实现摘帽脱贫，整体消除绝对贫困现象，决战全胜。这是我市发展史上具有重要意义的一件大事。全市上下特别是娄烦县要继续加大脱贫攻坚力度，从思想上拔“穷根”，从精准上做文章，从产业上夯基础，从成色上下功夫。加快光伏扶贫电站建设，推进马铃薯、中药材加工业，落实水源地生态补偿机制，全面完成易地扶贫搬迁，发展云顶山生态文化旅游，确保高质量脱贫摘帽。

实施乡村振兴战略。坚持规划先行，城乡融合，突出城郊型地域特色，推进产业振兴、人才振兴、文化振兴、生态振兴、组织振兴。坚持典型引领，结合水果、蔬菜、醋业、养殖等，促进农耕文化、地域文化和时尚文化相融合，发展休闲农业、农产品加工业和乡村旅游产业。坚持夯实基础，加强“三基”建设，改善农村水、电、路、通信等基础设施建设，完善农村生活设施，建设农民安居乐业的美丽家园，让良好生态环境成为乡村振兴的支撑点。

推进民生保障建设。全面完成五中、成成、一外、二外等新建工程。新改扩建公办幼儿园20所。加快职教园区规划建设。完成中心医院、人民医院、妇幼保健院等新建工程。加强教育、卫生人才队伍建设。加快城市棚户区改造建设，基本完成近年因重点工程涉拆群众安置回迁，落实拆迁惠民政策。加大城中村改造建设力度，启动30个城中村改造，已完成拆迁的村要全面加快建设。城镇棚户区改造新开工1.72万套，保障性安居工程基本建成5万套。继续加强公共停车场、公共厕所、人行天桥等建设，着力补齐民生短板。夯实县乡医疗卫生机构一体化改革基础，拓展家庭医生签约服务。大力发展养老服务产业，推动社区服务提档升级。完成老年福利院、儿童福利院、康宁医院等项目建设，给特殊群体更多的人性关怀和温暖。做好高校毕业生就业工作，实施全民技能提升工程。加快城乡居民基本医疗保险整合，城乡低保标准提高幅度不低于每人每月40元，发挥好社会保障的民生托底作用。

加强和改进社会治理。健全矛盾纠纷多元化解机制，妥善处理各类群体性事件。完善社会治安防控体系，依法开展扫黑除恶专项斗争。加强食品药品和农产品安全监管，确保老百姓“舌尖上的安全”。推进“双拥”和文明城市创建工作。坚决打击非法金融行为，防范化解重大风险。压实安全生产责任，确保社会和谐稳定。

（七）加强政府自身建设，打造人民满意的服务型政府

进入新时代，政府工作要有新气象新作为。要牢固树立“四个意识”，坚定“四个自信”，突出党的政治建设统领地位，坚决维护习近平总书记的核心地位，坚决维护党中央权威和集中统一领导，落实全面从严治党要求，加强自身建设，转变政府职能，为人民提供优质高效服务。

加快建设法治政府，全面推进依宪施政、依法行政。对宪法和法律始终保持敬畏之心，带头在宪法法律范围内活动，严格依照法定权限、规定、程序，行使权力，履行职责。按照中央和省、市委部署，有序推进机构改革。自觉接受人大及其常委会依法监督，接受人民政协民主监督，接受社会和舆论监督，认真听取民主党派、工商联、无党派人士和各人民团体意见。全面推进政务公开，让权力在阳光下运行，提高政府公信力。落实“谁执法谁普法”责任制，提高普法质量和效果。

时刻保持公仆本色，厚植各级干部“政德”之风。习近平总书记指出，政德是整个社会道德建设的风向标。立政德，就要明大德，铸牢理想信念，锤炼坚强党性，恪守立党为公；就要守公德，强化宗旨意识，增强政治定力，清清白白做人，干干净净干事；就要严私德，常思贪欲之害，常怀律己之心，心存敬畏，手握戒尺，严格操守，加强自律。

全面提高政府效能，增强政府公信力和执行力。路是走出来的，事是干出来的。人事有代谢，事业无止境。为人民干事是天职，不干是失职。广大干部要提高政治素质和工作本领，求真务实，干字当头；一分部署，九分落实；善始善终，善作善成。以真抓的实劲、敢抓的狠劲、善抓的巧劲、常抓的韧劲，多做打基础、利长远的好事，不计较个人进退得失，追求历史沉淀之后真正的评价。

各位代表，新时代是奋斗者的时代。团结凝聚力量，实干创造未来。让我们更加紧密地团结在以习近平同志为核心的党中央周围，高举习近平新时代中国特色社会主义思想伟大旗帜，不忘初心、牢记使命，锐意进取、顽强拼搏，奋力谱写文明开放富裕美丽太原新篇章！
